# Development of Real-Time Kinematic Magnetic Resonance Imaging (kMRI) Techniques for Studying the Kinematics of the Spine and Joints in Dogs—Preliminary Study on Cadavers

**DOI:** 10.3390/ani12202790

**Published:** 2022-10-15

**Authors:** Sara Canal, Roberto Tamburro, Ilaria Falerno, Francesca Del Signore, Francesco Simeoni, Francesco De Pasquale, Andrea De Bonis, Annamaria Maraone, Andrea Paolini, Amanda Bianchi, Martina Rosto, Massimo Vignoli

**Affiliations:** Faculty of Veterinary Medicine, Veterinary Teaching Hospital, University of Teramo, 64100 Teramo, Italy

**Keywords:** real-time kinematic MRI, canine, cervical spine, elbow, stifle

## Abstract

**Simple Summary:**

Many orthopedic and neurological pathologic conditions can potentially lead to or be affected by joint instability. Standard magnetic resonance imaging, as a static technique that examine joints and body parts in functional rest, can underestimate or overlook key diagnostic findings. As a result, kinematic magnetic resonance imaging techniques were developed to evaluate joints and body parts under stress and load conditions or during movement. In human medicine, the real-time acquisition technique is one of the modalities for acquiring kinematic magnetic resonance imaging, and has gained popularity in recent years. This proof-of-concept study was designed to test the feasibility of real-time acquisition techniques in veterinary medicine for the first time. Based on the results of this preliminary cadaveric study, real-time kinematic magnetic resonance imaging may be a feasible and valuable procedure to be applied to the canine cervical spine and stifle joints. Moreover, given the ease of execution and the concise duration of acquisitions, it could be applied in a regular standard protocol MRI with little additional effort, risk, and cost. In this proof-of-concept study, a good visualization of the canine cervical spine and stifle joint was achieved, showing the potential of real-time acquisition techniques for clinical and research applications.

**Abstract:**

Kinematic MRI (kMRI) is a novel human imaging technique that couples the excellent soft tissue contrast and multiplanar capabilities of traditional MRI with kinematic potential. The study’s goals are: (1) testing the feasibility of spinal cord and joints real-time kMRI; and (2) evaluating the quality of these kinematic studies as a new diagnostic option in veterinary medicine. Standard and real-time kinematic MRI were performed on cervical spine, elbow, and stifle joints of seven cadavers. Studies were repeated after a surgical insult aimed to create a certain degree of joint instability. A total of 56 MRI were performed—7 cervical spinal tracts, 3 elbow joints, and 4 stifle joints were examined. The technique was feasible in all the three regions examined. The images were considered of excellent quality for the stifle joint, good to fair for the cervical spine, whereas two of three elbow studies were considered to have unacceptable image quality. Additionally, real-time kMRI provided good to excellent information about stifle instability. Therefore we consider kMRI a promising technique in veterinary medicine. Further studies and an in vivo setting are needed to increase the quality of the kMRI images, and to fully evaluate clinical usefulness.

## 1. Introduction

Kinematics is a branch of mechanics covering the study of motion. Traditional kinematic gait analyses allow a proper assessment of joint function and biomechanics, objectively assessing joint motion characteristics, such as range of motion, acceleration, and velocity [1,2]. However, the interpretation of these data poses significant limitations in identifying the intrinsic factors contributing to joint dysfunction and abnormal motion. Magnetic resonance imaging is an excellent advanced imaging tool for the study of both orthopedic [3,4,5] and neurological diseases [6,7]. Due to its high soft tissue contrast, musculoskeletal structures (i.e., tendons, ligaments, fascial and synovial membranes), nervous structures, blood vessels, and osseous tissues can be clearly assessed and evaluated for pathological changes [3,4,5,6,7,8]. Conventional MRI studies are usually performed at functional rest. However, these static studies can potentially underestimate the disease entity and fail to characterize the dynamic physiology of joints [9,10]. As a result, kMRI is performed, imaging patients in weight-bearing or stressed positions [9,10]. In human medicine, three kMRI methods exist: (1) incremental (quasi-static) acquisition, i.e., a static MRI study repeated after a change in the joint/spine position; (2) motion-triggered acquisition, i.e., cine-MRI and cine phase contrast techniques applied while cycles of joint movement occur; and (3) real-time acquisition, i.e., few seconds real-time MRI acquisition during a single joint movement [10]. In veterinary medicine, kMRI studies are not routinely performed in clinical settings. However, several studies have been published highlighting its usefulness in diagnosing both neurological and orthopedical diseases in and ex vivo settings [11,12,13,14,15,16,17]. All these studies analyzed the feasibility and utility of incremental acquisition kMRI techniques. One major drawback of this method is the considerable amount of time added to the standard MRI protocol when used in a clinical setting, consequently increasing the anesthetic time and risk for the patient, as well as the overall exam time and cost [12,14]. The real-time acquisition technique is a very fast kMRI modality allowing the study of joint and spine movements in a few seconds [10,18,19]. To the authors’ knowledge, this method has never been tested in veterinary medicine. Thus, the objectives of this proof-of-concept study were: (1) to test the feasibility of spinal cord and joint kMRI by use of real-time acquisition techniques in canine cadavers, and (2) to evaluate the quality of real-time acquisition kMRI studies as a new diagnostic option in veterinary medicine.

## 2. Materials and Methods

### 2.1. Subjects

Patient selection and data acquisition were performed between January 2022 and May 2022. The study was conducted on 7 canine cadavers who died for reasons unrelated to neurological and orthopedic diseases. These 7 dogs included 2 females and 5 males, all entire, ranging in age from 6 to 12 years. Breeds included 2 mongrels (1 extra-small and 1 large), 1 French Bulldog, 1 Dobermann Pinscher, 1 Pitbull, 1 Rottweiler, and 1 Weimaraner (Table 1). Each breed was assigned to a size category using the mean adult weight listed by the American Kennel Club, as previously reported [20]. Breeds were classified as extra small (≤6.8 kg), small (>6.8 kg, ≤13.6 kg), medium (>13.6 kg, ≤25 kg), large (>25 kg, ≤38.6 kg), or giant (>38.6 kg).

Cadavers were collected and stored at −5 °C within 12 h following euthanasia. Dogs were thawed at room temperature of 20 °C in preparation for the study. 

An inclusion criterion to be met was the absence of any lesion involving soft tissues and bones in the regions of interest (i.e., the cervical region of the spine, elbows, and stifles).

### 2.2. Instrumentation and Protocol

**Figure 1:**
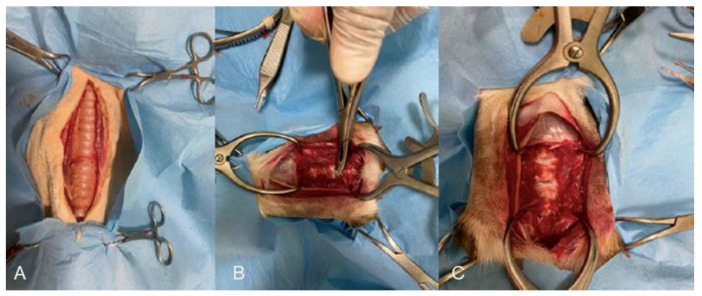
(**A**) Before, (**B**) during, and (**C**) after the ventral slot procedure to remove two intervertebral disks and the dorsal longitudinal ligament.

All scans were acquired using a low-field open-bore MRI unit (Esaote, VET-MR GRANDE, 0.25 T, Genoa, Italy). A static and kinematic MRI study was conducted for each region scanned, before and after a surgical insult was applied to create a certain degree of articular instability. Static MRI protocol included T1-weighted (T1W) and T2-weighted (T2W) images acquired in both sagittal and transverse planes. Kinematic MRI protocol included a real-time acquisition during one cervical spine, elbow, and stifle motion cycle. The real-time kMRI sequence was a custom-built gradient-echo “steady-state” balanced type sequence (2D HYCE S) acquired in the sagittal plane. Dogs were placed in dorsal recumbency while scanning the cervical region by use of a fast gradient elliptical coil and in lateral recumbency while scanning elbow and stifle regions by use of a fast gradient dual phase array elliptical coil. Pre-surgical injury static MRI studies were evaluated to certify the absence of any pathological abnormalities. At the same time, post-surgical injury static MRI studies were acquired to confirm expected pathological changes. During kMRI acquisition, an operator (A.M.) performed passive movements of the anatomical region scanned as follows: a) flexion and extension movements for the cervical spine, elbow, and stifle region; and b) tibial compression test (TCT) for the stifle region. The surgical insult applied to the cervical spine was performed by a ventral approach following a previously described technique (ventral slot procedure) [21] modified to entirely remove two intervertebral disks in the C4–C7 cervical region and the dorsal longitudinal ligaments (Figure 1). Regarding the surgical insult applied to the elbow joint, the cadaver was placed in lateral recumbency with the affected leg down and the uppermost leg caudally retracted. A medial skin incision was performed starting from the medial epicondyle to the proximal radius. A Gelpi retractor allowed the separation of the flexor carpi radialis with the pronator teres muscles to visualize the medial collateral ligament and the joint capsule that were totally transected using a #11 scalpel blade. Then, the soft tissues were routinely closed [22]. The surgical insult applied to the stifle consists of a craniomedial parapatellar skin incision to approach the stifle joint starting from the proximal aspect of the patella to the tibial tuberosity. A 2–3 cm medial stifle arthrotomy was performed using a #11 scalpel blade. A Gelpi retractor and stifle distractors were respectively applied in medio-lateral and proximo-distal directions. Then, the cranial cruciate ligament was visualized and totally transected using a #11 scalpel blade. The joint capsule and the soft tissues were routinely closed [23]. 

### 2.3. Image Analysis 

Images were analyzed by an ECVDI (i.e., European College of Veterinary Diagnostic Imaging) board-certified radiologist (M.V.), an ECVN (i.e., European College of Veterinary Neurology) board-certified neurologist (S.C.), and a first year ECVDI resident (A.D.B.). The final decision on the imaging characteristics was reached on a consensus basis.

All the studies were reviewed using a DICOM (i.e., Digital Imaging and Communications in Medicine) freely available viewer (Horos, Horosproject.org, Nimble Co LLC d/b/a Purview in Annapolis, MD, USA). The three observers were asked to fill in a predefined standardized commercially available spreadsheet (Microsoft Excel 2020, Microsoft, Redmond, WA, USA). In all kMRI studies, observers were asked to subjectively evaluate: (1) the presence of artifacts (score 0, unacceptable artifacts precluding evaluation of images; score 1, acceptable artifacts not precluding evaluation of images), (2) the quality of the passive motion applied (score 0, fast, irregular, and not fluid; score 1, adequate fluid and constant movement), (3) maintenance of a mid-sagittal plane during the entire sequence (score 0, not maintained; score 1, maintained), (4) sharpness and quality definition of the images (score 0, blurry and ill-defined images; score 1, sharp and well-defined images). The overall final score, derived from the evaluation of all 4 criteria, was as follows: unacceptable results (overall score 0 and 1), fair results (overall score 2), good results (overall score 3), and excellent results (overall 4). Additionally, post-surgical injury kMRI studies were subjectively assessed for evidence of articular instability. Two intervertebral spaces (between C4–C5 and C6–C7) were evaluated for subluxation; vertebral canal stenosis and spinal cord extradural compression were regarded as evidence of vertebral instability in the cervical spine. Joint space widening was regarded as evidence of elbow joint instability. Joint space widening and cranial subluxation of the tibia were regarded as evidence of stifle joint instability. 

## 3. Results

A total of 56 MRI studies were evaluated (14 pre-surgical injury static MRI, 14 post-surgical injury static MRI, 14 pre-surgical injury kMRI, and 14 post-surgical injury kMRI). Overall, 7 cervical spine (all cases), 4 stifles (Case 6 and Case 7 bilaterally) and 3 elbows (Case 5 bilaterally and Case 6 right elbow) images were collected. The median time to evaluate a single region (2 static MRI and 2 kMRI), including the surgical procedure, was 4 h (range 3–5 h). Overall kMRI scores are presented in Table 2. 

### 3.1. Cervical Spine kMRI

**Figure 2:**
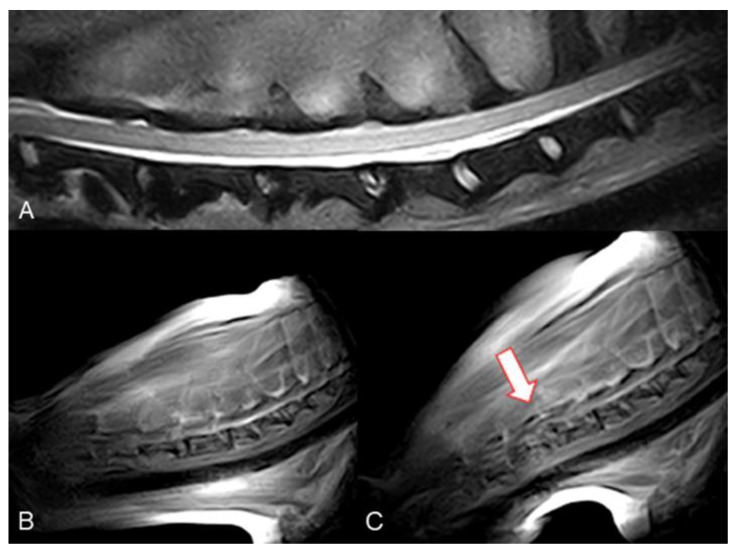
Mongrel, 7 years old, male entire (Case 4). Cervical spine MRI sample images. (**A**) T2 weighted mid-sagittal image of the cervical spine. (**B**) Real-time kMRI starting position, no spinal cord compression is visible. (**C**) Real-time kMRI during flexion movement, C4–C5 mild subluxation is evident (arrow).

**Figure 3:**
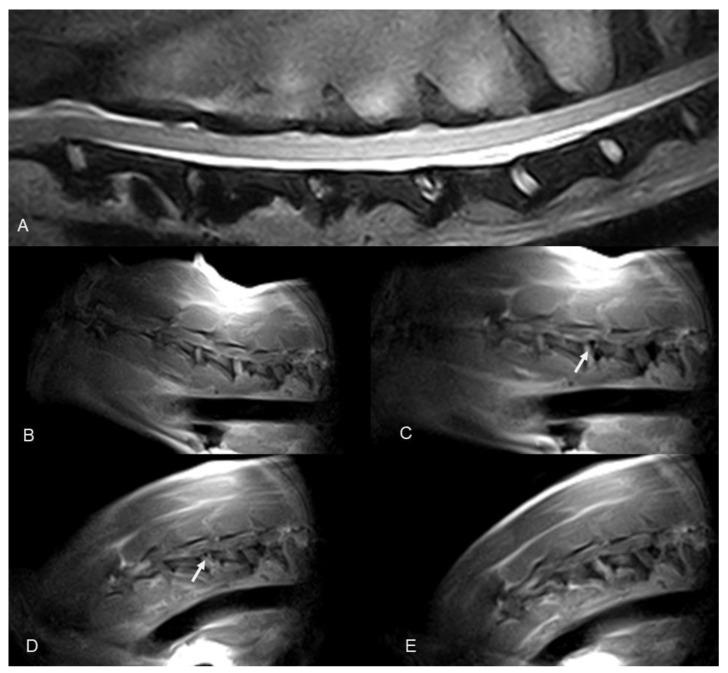
Dobermann, 8 years old, male entire (Case 7). Cervical spine MRI sample images: (**A**) T2 weighted mid-sagittal image of the cervical spine. (**B**–**E**) Real-time kMRI during neck flexion, C4–C5 mild dynamic subluxation is visible (arrows).

The overall evaluation of cervical spine kMRI was fair to good. Case 1 to Case 4 kMRI were our first four attempts at performing real-time kinematic acquisitions. Unfortunately, passive movement of the spine was inadequate (too rapid and not fluid), causing artifacts that precluded the evaluation of the images, and loss of the midsagittal plane and image quality definition. Moreover, Case 2 kMRI quality of the study was also impaired by the small subject size, thus decreasing further image quality. After the first four attempts, we decided to perform the cycle of motion in about 40–60 s; in all these studies, the quality of movement was considered adequate, and motion artifacts decreased substantially. Thus, all three additional cases obtained real-time acquisitions that were evaluated as good. In all these three post-surgical injury real-time kMRI studies, vertebral canal stenosis due to post-surgical vertebral instability was subjectively confirmed during extension and flexion movements; however, clear evidence of spinal cord compression was lacking due to the high imaging definition of vertebral structures but low spinal cord imaging definition (Figure 2 and Figure 3, Supplementary Material Video S1 and S2).

### 3.2. Elbow kMRI

**Figure 4:**
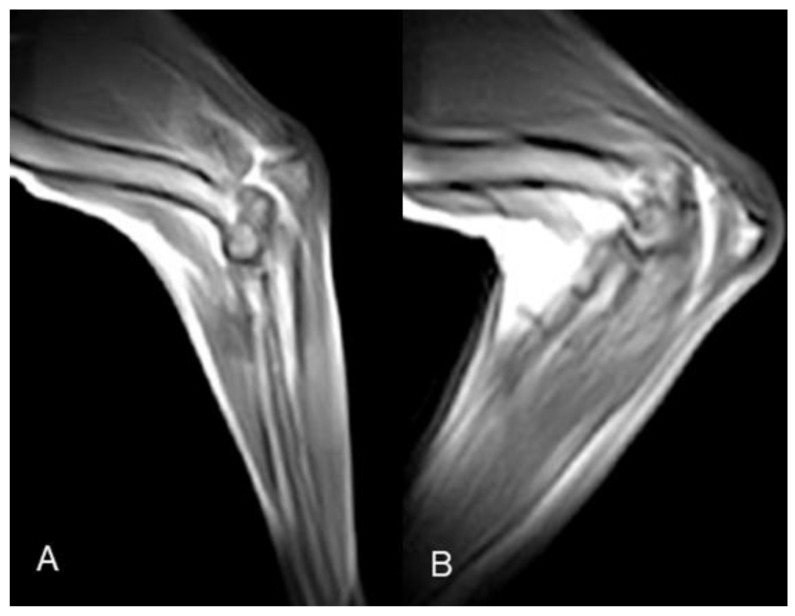
Case 6 elbow k-MRI sample images. (**A**) Neutral starting position; (**B**) point of maximum flexion.

The evaluation of elbow kMRI studies was unacceptable in Case 5 (both sides), our first attempts at this region. The passive movement applied to the elbow was inadequate (too rapid and not fluid), causing artifacts and the variation of a constant mid-sagittal plane. The overall evaluation of Case 6 elbow kMRI study was good. The passive movement was improved, constantly fluid, and slower than the previous two attempts. Thus, artifacts decreased, image quality improved, and the mid-sagittal plane was overall retained. In all three post-surgical injury kMRI studies, articular instability was not evident during extension and flexion movements. The duration of the 2D HYCE S in Case 6, namely the time required for a single cycle of elbow flexion and extension, was 20–25 s (Figure 4, Supplementary Material Video S3).

### 3.3. Stifle kMRI

**Figure 5:**
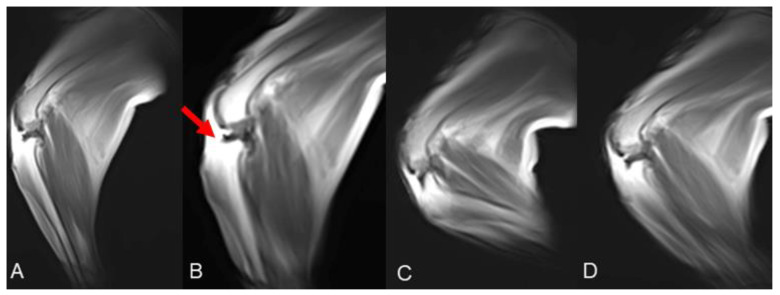
Case 6 stifle k-MRI sample images. (**A**) Starting position, normal femorotibial alignment. (**B**) TCT endpoint, cranial subluxation of the tibia is visible, due to surgical cranial cruciate ligament resection; (**C**) flexion and (**D**) extension movement, femorotibial, no subluxation visible.

All stifle kMRI studies were evaluated as excellent. The passive movement, substantially improved after cervical and elbow kMRI studies, was constantly fluid and slow. Thus, artifacts decreased, and the mid-sagittal plane was overall retained. The duration of the 2D HYCE S, namely the time required for a single cycle of stifle flexion and extension, was 20–25 s. In all four post-surgical injury kMRI studies, articular instability was evident during TCT movements (Figure 5, Supplementary Material Video S4).

## 4. Discussion

Based on the results of this proof-of-concept preliminary cadaveric study, the real-time acquisition technique is a feasible kMRI procedure that can be applied to the canine cervical spine, elbow and stifle joints.

Among the currently available diagnostic imaging techniques, MRI can provide the greatest range of information and the finest accuracy in detecting subtle abnormalities due to the highest soft tissue contrasts [3,4,5,6,7,8,9,10]. Conventional MRI studies are conducted at functional rest, but some spinal or orthopedical diseases, characterized by articular instability and a dynamic component, can be underestimated when imaged in these conditions [10]. As a result, kMRI is performed, imaging patients in weight-bearing or stressed positions [9,10]. In veterinary medicine, the kMRI diagnostic potential has already been demonstrated in both ex vivo and in vivo settings. Disc- and osseous-associated cervical spondylomyelopathy, tethered cord syndrome, and degenerative lumbosacral stenosis are some of the neurological diseases that can benefit from the diagnostic improvement of the kMRI [12,13,14,15,16,17]. Kinematic MRI allows the visualization of spinal cord and cauda equina compressions not identified in conventional static MRI protocols [12,14,16]. Additionally, kMRI led to a diagnosis of tethered cord syndrome in a dog demonstrating decreased craniocaudal displacement of the conus medullaris in flexed and extended positions [13]. Among orthopedic diseases, cranial cruciate ligament intact and deficient stifle joints were imaged under stressed conditions in an ex vivo setting, highlighting the potential to measure and diagnose stifle joint subluxation [11].

In human medicine, kMRI studies are conducted with both low- and high-field MRI units [9,10]. Open-bore scanners are more adaptable to perform these dynamic studies, specifically when it is paramount to evaluate the spine or joints in a weight-bearing position (upright scanning) or those pain that are position or movement-dependent [9,24]. However, the drawback of open magnet scanners lies in the lower field strength, which results in decreased signal-to-noise ratio and thus lower resolution of the studies [24]. In our study, all kMRI was performed with a low-field open-bore MRI unit (0.25 T), and the images acquired were consequently characterized by a lower resolution compared to human high-field real-time kMRI studies [25,26].

In human medicine, real-time kMRI studies are conducted either allowing patients to perform a self-selected rate of motion [25,27] or by using an MR compatible positioning device that guides and standardizes patient movement [28]. A significant difference between human and veterinary real-time kMRI studies is the human patient’s possibility to perform one cycle movement voluntary, whereas this would not be the case in our anaesthetized patients. Thus, the necessity in veterinary real-time kMRI studies is an operator performing the maneuver. In our study, all the movements were performed freely without using any devices, and were only limited by the free space inside the coil and scanner bore. Although we are aware that the characteristics of this type of movement introduce potential variability, it has already been demonstrated in human medicine that differences between different rates and types of motion do not affect overall results [27]. Further studies evaluating the difference between real-time kMRI sequences with cycle motions acquired with or without a guiding device are also needed to confirm this hypothesis in veterinary medicine.

In human medicine, optimal real-time kMRI of the joints and spine requires three criteria to be accomplished: (1) available dynamic MRI sequences with an acquisition time as fast as possible to image the region of interest during a single cycle of voluntary movement; (2) customized scanning parameters and MRI sequences according to the field strength and manufacturer of the MRI machine; (3) standard radiofrequency coil positioning and patient setup for each region of interest and its range of motion [10]. Regarding the first criterion of a real-time kMRI fast imaging technique, rapid gradient-echo sequences are frequently used due to their high temporal resolution, permitting a single image acquisition in a few hundred milliseconds and fast repetition of the slices [10]. In a recent study of real-time 3-T kMRI assessment of spine kinematics, all the fast imaging sequences were acquired in less than 77 s, nearly 5% of the overall scan duration [25]. In our study, a 2D HYCE S sequence was used during the acquisition of the dynamic MRI. This particular sequence is a gradient echo “steady-state” balanced type sequence that enables continuously repeated acquisition of images of the same slice, set by the user. Based on the parameters the user selects (TR, number of acquisitions, series, and phases) high-speed acquisition is possible up to approximately one image (frame) per second. In our study, after an initial training, we set the duration of the 2D HYCE S sequence during one motion cycle of flexion and extension of the stifle and elbow at about 20–25 s, whereas the duration of the sequences in the spine was around 40–60 s. Performing the cycle of motion in that interval time allowed us to obtain good quality images with a substantially limited amount of motion artifacts. Therefore, compared to the kMRI modalities previously investigated in veterinary medicine, this kinematic technique is more advantageous and affordable. Provencher et al. evaluated the use of high-field kMRI in canine osseous-associated [14] and disc-associated [12] cervical spondylomyelopathy. Although the technique was considered feasible and provided additional diagnostic information beyond the static MRI study, a significant drawback was the overall considerable amount of time added to the traditional MRI protocol [12,14]. This aspect is of considerable importance given the increased cost for the owner and anesthesiologic risk for the patient. Therefore, as for human medicine, we could consider real-time kMRI a superior kinematic technique in terms of acquisition time, costs, and potential anesthetic risks compared to incremental acquisitions.

An additional significant aspect to be considered in human kMRI studies is the standardization of radiofrequency coil positioning and patient setup for each region of interest and its range of motion [10]. Although kMRI is widely diffuse and recognized for its relevant diagnostic properties, there is still no consensus regarding technical parameters and setups [10,19]. This aspect could probably relate to the presence of multiple and custom-made kinematic sequences and various original equipment manufacturers for the MRI scanners [10,28]. In our study, we used a custom-calibrated elliptical coil, cadavers placed in dorsal recumbency for cervical spine evaluation, and lateral recumbency for stifle and elbow evaluation. The scanner’s characteristics (open-bore) and the coils provided enough space to allow our operator to perform all cycles of passive movements in all three regions of interest. Additionally, there was no need to change the recumbency of the cadaver to allow the movement to be performed. In human medicine, real-time kMRI studies are conducted in both open- and closed-bore scanners. However, as canine patients have to be anesthetized during imaging studies, we think that real-time veterinary kMRI studies could be less feasible in a closed-bore MRI scanner. Due to space limitations, these MRI machine types prevent an operator from comfortably performing one movement cycle. Moreover, in the two high-field kMRI studies available for the cervical spine, the authors needed to change the recumbency of their patient (from dorsal to lateral) after the standard MRI protocol in a neutral position [12,14]. The highlighted shortcomings of this technique were as follows: (1) labor-intensive positioning of the patients; (2) inability to standardize the degrees of flexion and extension due to dogs’ size variation and space available inside the closed-bore scanner, and (3) difficulty in imaging resetting after the recumbency change. In our cervical real-time kMRI studies, changing cadavers and radiofrequency coil positioning was not needed after the static MRI protocol. In the authors’ opinion, this is another significant advantage of real-time kinematic techniques that leads to shortened protocol time, superior ease of execution, and no need to reset sequence parameters to adapt to the change in patient recumbency. Regarding canine stifle kMRI, only one ex-vivo study has been published so far [11]. Although the authors confirmed the diagnostic utility of this technique in objective identification and measurement of femorotibial subluxation in cranial cruciate deficient stifle joint, no further in vivo studies have been published. The kinematic technique investigated in Tremolada et al. research [11] was an incremental acquisition performed by using of an MRI-compatible stifle-loading jig. As in human medicine, the main limitation of these techniques is the lack of availability and reproducibility of such devices by other groups [19,28]. Furthermore, to allow proper positioning, a bone tunnel was drilled, and two Kirschner wires were placed in each cadaver’s tibia. Thus authors declare that this technique could not be reproduced in clinical settings [11]. In our study, the passive movement of the joint was easily feasible in all stifles, and, as these kMRI studies were one of the last to be acquired, a considerable improvement in the adequate movement technique was achieved, leading to 8/8 (pre- and post-surgical injury) excellent scores. Furthermore, although not the primary study goal, in all four real-time post-surgical insult kMRI studies, an instability of the joint was subjectively easily assessed.

To the authors’ knowledge, this is the first study to apply kMRI to the canine elbow joint. To date, few studies have described the use of MRI to evaluate elbow diseases such as medial compartment disease [29], incomplete ossification of the humeral condyle [30,31], or elbow dysplasia [32]. Furthermore, evidence of the clear superiority of standard MRI compared with other diagnostic modalities as arthroscopy is lacking [29]. In our study, elbow real-time kMRI technique was feasible, but image acquisitions were considered of unacceptable quality in two of three cases. Possible explanations for the lower accuracy of dynamic elbow studies could be as follows: (1) the lower numbers of examinations performed precluding proper training and settings; (2) previously mentioned intrinsic joint factors, such as relatively small size and complex articulations [33]; and (3) larger free space between the elbow and coil surface that could decrease the signal-to-noise ratio [34]. Further studies with more significant case numbers are needed to confirm these results.

This study has several limitations. The ex vivo research concept means that the real-time kMRI technique fails to accurately replicate the complexities of joints and spine since cadaveric studies do not imitate in vivo conditions [24]. To minimize tissue autolysis, the cadaver were frozen within 12 h of death and were subsequently thawed at an ambient temperature of 20 °C to reduce cellular damage from a slow thawing process. However, given the overall duration of the MRI protocol (2 static MRIs and 2 real-time kMRIs + surgical time) for each region of interest and the acquisition of more than one MRI protocol in three instances (Case 5, Case 6, and Case 7), we have to consider that image quality could decrease due to tissue deterioration despite our efforts to minimize it. Further research, possibly with the aim of reducing protocol time in ex vivo settings, is needed. The optimization of real-time kMRI studies and translation into an in vivo setting was not the objective of this study and should be the subject of future works. Another limitation could be the operator’s role in performing the movement that could not be easily standardized and objectively and constantly reproduced. A training period could be advisable to perform adequate slow and in-line movement to minimize motion artifacts and to maintain the sagittal plane, thus permitting satisfactory image quality. In general, we noticed that the first kMRI studies were of poorer quality, with a progressive improvement during the development of the study. Obviously, a period of training is necessary to acquire the correct manual skills, both for the speed of movement and the maintenance of the axis of the structure to be studied. As previously documented in veterinary medicine, kMRI could be indicated in the overall diagnostic evaluation of diseases such as caudal cervical spondylomyelopathy [12,14], degenerative lumbosacral stenosis [15,16,17], and joint instability [11]. Future studies are needed to evaluate clinical impact of real-time kMRI acquisitions in the diagnosis of those diseases. Another interesting field of application could be the evaluation of shoulder instabilities, as already demonstrated in human medicine [35].

## 5. Conclusions

Based on the results of this preliminary cadaveric study, the real-time acquisition technique could be a feasible and valuable kMRI procedure to be applied to the canine cervical spine and stifle joints. Further studies are needed to evaluate its utility in elbow joint MRI studies. A real-time kMRI technique could suggest the ability to acknowledge in-vivo joint kinematics and, consequently, diagnose dynamic pathologies such as joint and spine instability syndromes. Given the ease of execution and the very short acquisition time, real-time kMRI studies could be potentially applied in a regular standard protocol MRI with limited additional efforts, risks, and costs, providing attractive alternatives to previous kMRI techniques. In this proof-of-concept study, a good visualization of canine cervical spine and stifle joint could be achieved, showing the potential of real-time kMRI for clinical and research applications.

## Figures and Tables

**Table 1:** Signalment of the 7 dogs.

Case	Breed	Sex ^1^	Age ^2^	Breed Size
Case 1	Weimaraner	ME	9	Large
Case 2	Mongrel	ME	12	Extra small
Case 3	French Bulldog	FE	11	Small
Case 4	Mongrel	ME	7	Large
Case 5	Rottweiler	FE	6	Giant
Case 6	Pitbull	ME	8	Medium
Case 7	Dobermann Pinscher	ME	8	Large

^1^ ME, male entire; FE, female entire; ^2^ Age in years.

**Table 2:** Overall kMRI scores (pre- and post-surgical injury).

Case	Cervical Spine	Elbow ^1^	Stifle ^2^
Case 1	fair	N.A.	N.A.
Case 2	fair	N.A.	N.A.
Case 3	fair	N.A.	N.A.
Case 4	fair	N.A.	N.A.
Case 5	good	unacceptable (RE) unacceptable (LE)	N.A.
Case 6	good	good (RE)	excellent (RS) excellent (LS)
Case 7	good	N.A.	excellent (RS) excellent (LS)

^1^ RE right elbow, LE left elbow; ^2^ RS right stifle, LS left stifle.

## Data Availability

The data presented in this study are available on request from the corresponding author.

## Supplementary Materials

The following supporting information can be downloaded at: https://www.mdpi.com/article/10.3390/ani12202790/s1, Video S1: cervical spine real-time kMRI; Video S2: cervical spine real-time kMRI; Video S3: elbow real-time kMRI; Video S4: stifle real-time kMRI.

## References

[1] Decamp C.E. (1997). Kinetic and kinematic gait analysis and the assessment of lameness in the dog. Vet. Clin. N. Am. Small Anim. Pract..

[2] Millis D., Janas K. (2021). Forelimb Examination, Lameness Assessment, and Kinetic and Kinematic Gait Analysis. Vet. Clin. N. Am. Small Anim. Pract..

[3] Gavin P.R., Holmes S.P., Gavin P.R., Bagley R.S. (2009). Orthopedic. Practical Small Animal MRI.

[4] Adamiak Z., Jaskólska M., Matyjasik H., Pomianowski A., Kwiatkowska M. (2011). Magnetic resonance imaging of selected limb joints in dogs. Pol. J. Vet. Sci..

[5] Sage J.E., Gavin P. (2016). Musculoskeletal MRI. Vet. Clin. N. Am. Small Anim. Pract..

[6] Dennis R. (2011). Optimal magnetic resonance imaging of the spine. Vet. Radiol. Ultrasound.

[7] Robertson I. (2011). Optimal magnetic resonance imaging of the brain. Vet. Radiol. Ultrasound.

[8] Przeworski A., Adamiak Z., Głodek J. (2016). Comparison of High-field and Low-field Magnetic Resonance Imaging of Stifle Joint Disorders in Dogs. Pol. J. Vet. Sci..

[9] Michelini G., Corridore A., Torlone S., Bruno F., Marsecano C., Capasso R., Caranci F., Barile A., Masciocchi C., Splendiani A. (2018). Dynamic MRI in the evaluation of the spine: State of the art. Acta Biomed..

[10] Garetier M., Borotikar B., Makki K., Brochard S., Rousseau F., Ben Salem D. (2020). Dynamic MRI for articulating joint evaluation on 1.5 T and 3.0 T scanners: Setup, protocols, and real-time sequences. Insights Imaging.

[11] Tremolada G., Winter M.D., Kim S.E., Spreng D., Pozzi A. (2014). Validation of stress magnetic resonance imaging of the canine stifle joint with and without an intact cranial cruciate ligament. Am. J. Vet. Res..

[12] Provencher M., Habing A., Moore S.A., Cook L., Phillips G., da Costa R.C. (2016). Kinematic Magnetic Resonance Imaging for Evaluation of Disc-Associated Cervical Spondylomyelopathy in Doberman Pinschers. J Vet. Intern. Med..

[13] De Decker S., Watts V., Neilson D.M. (2017). Dynamic Lumbosacral Magnetic Resonance Imaging in a Dog with Tethered Cord Syndrome with a Tight Filum Terminale. Front. Vet. Sci..

[14] Provencher M., Habing A., Moore S.A., Cook L., Phillips G., da Costa R.C. (2017). Evaluation of osseous-associated cervical spondylomyelopathy in dogs using kinematic magnetic resonance imaging. Vet. Radiol. Ultrasound.

[15] Zindl C., Tucker R.L., Jovanovik J., Gomez Alvarez C., Price D., Fitzpatrick N. (2017). Effects Of Image Plane, Patient Positioning, And Foraminal Zone On Magnetic Resonance Imaging Measurements Of Canine Lumbosacral Intervertebral Foramina. Vet. Radiol. Ultrasound.

[16] Lampe R., Foss K.D., Hague D.W., Oliveira C.R., Smith R. (2020). Dynamic MRI is reliable for evaluation of the lumbosacral spine in healthy dogs. Vet. Radiol. Ultrasound.

[17] Nam J., Kang K., Kim K., Choi J., Choi M., Yoon J. (2021). Translocation of the conus medullaris during dynamic lumbosacral magnetic resonance imaging in dogs. Am. J. Vet. Res..

[18] Quick H.H., Ladd M.E., Hoevel M., Bosk S., Debatin J.F., Laub G., Schroeder T. (2002). Real-time MRI of joint movement with trueFISP. J. Magn. Reson. Imaging.

[19] Walter W.R., Burke C.J. (2022). Editorial Commentary: Real-Time Dynamic Magnetic Resonance Imaging of the Patellofemoral Joint: Ready for Prime Time?. Arthroscopy.

[20] Greene E., Rendahl A., Goldschmidt S. (2022). The anatomical relationship between the mandibular first molar roots and the mandibular canal based on breed size and skull type. Front. Vet. Sci..

[21] Sharp N.J., Wheeler S.J., Sharp N.J., Wheeler S.J. (2005). Cervical disc disease. Small Animal Spinal Disorders: Diagnosis and Surgery.

[22] Fitzpatrick N., Smith T.J., Evans R.B., O’Riordan J., Yeadon R. (2009). Subtotal Coronoid Ostectomy for Treatment of Medial Coronoid Disease in 263 Dogs. Vet. Surg..

[23] Piermattei D.L., Johnson K.A., Piermattei D.L., Johnson K.A. (2004). Approach to the stifle joint through medial incision. An Atlas of Surgical Approaches to the Bones and Joints of the Dog and Cat.

[24] Shapiro L.M., Gold G.E. (2012). MRI of weight bearing and movement. Osteoarthr. Cartil..

[25] Walter W.R., Alizai H., Bruno M., Portugal S., Burke C.J. (2021). Real-time dynamic 3-T MRI assessment of spine kinematics: A feasibility study utilizing three different fast pulse sequences. Acta Radiol..

[26] Frings J., Dust T., Krause M., Frosch K.H., Adam G., Warncke M., Welsch G., Henes F.O., Maas K.J. (2022). Dynamic Mediolateral Patellar Translation Is a Sex- and Size-Independent Parameter of Adult Proximal Patellar Tracking Using Dynamic 3 Tesla Magnetic Resonance Imaging. Arthroscopy.

[27] d’Entremont A.G., Nordmeyer-Massner J.A., Bos C., Wilson D.R., Pruessmann K.P. (2013). Do dynamic-based MR knee kinematics methods produce the same results as static methods?. Magn. Reson. Med..

[28] Gerigk L., Bostel T., Hegewald A., Thomé C., Scharf J., Groden C., Neumaier-Probst E. (2012). Dynamic magnetic resonance imaging of the cervical spine with high-resolution 3-dimensional T2-imaging. Clin. Neuroradiol..

[29] Franklin S.P., Burke E.E., Holmes S.P. (2017). Utility of MRI for Characterizing Articular Cartilage Pathology in Dogs with Medial Coronoid Process Disease. Front. Vet. Sci..

[30] Gabriel P., Pfeil A., Ludewig E., Böttcher P., Oechtering G. (2009). Magnetic resonance imaging diagnosis: Incomplete ossification of the humeral condyle in a German shepherd dog. J. Small Anim. Pract..

[31] Piola V., Posch B., Radke H., Telintelo G., Herrtage M.E. (2012). Magnetic resonance imaging features of canine incomplete humeral condyle ossification. Vet. Radiol. Ultrasound.

[32] Reichle J.K., Snaps F. (1999). The elbow. Clin. Tech. Small. Anim. Pract..

[33] Cook C.R., Cook J.L. (2009). Diagnostic imaging of canine elbow dysplasia: A review. Vet. Surg..

[34] Zalcman A.R., Cook C., Mai W., Mai W. (2018). General Features and Optimized Technique for the Musculoskeletal System. Diagnostic MRI in Dogs and Cats.

[35] Tempelaere C., Pierrart J., Lefèvre-Colau M.M., Vuillemin V., Cuénod C.A., Hansen U., Mir O., Skalli W., Gregory T. (2016). Dynamic Three-Dimensional Shoulder Mri during Active Motion for Investigation of Rotator Cuff Diseases. PLoS ONE.

